# Recent Insights Into Sleep Paralysis: Mechanisms and Management

**DOI:** 10.7759/cureus.65413

**Published:** 2024-07-26

**Authors:** Vijay Bhalerao, Shashank Gotarkar, Deepak Vishwakarma, Sushim Kanchan

**Affiliations:** 1 Department of Community Medicine, Jawaharlal Nehru Medical College, Datta Meghe Institute of Higher Education & Research, Wardha, IND

**Keywords:** rapid eye movement (rem) sleep, treatment modalities, risk factors, hallucinations, sleep paralysis

## Abstract

Sleep paralysis (SP) is a phenomenon wherein individuals awaken from deep sleep but are unable to move or speak, often experiencing vivid hallucinations. This condition, attributed to the persistence of muscle atonia from rapid eye movement (REM) sleep into wakefulness, is associated with factors like sleep deprivation and irregular sleep patterns. While isolated episodes of SP are generally benign, recurrent episodes may warrant clinical attention, particularly when accompanied by distressing symptoms. Despite its prevalence across cultures and its documented association with various medical conditions, SP remains poorly understood by many. This review explores the clinical characteristics, epidemiology, and associated risk factors of SP, drawing from a comprehensive analysis of the existing literature. Additionally, the review discusses potential treatment modalities, including pharmacological interventions and cognitive-behavioral therapy, highlighting the need for further research to enhance our understanding and management of this intriguing phenomenon.

## Introduction and background

Sleep paralysis (SP) is when an individual awakens from a deep sleep yet cannot move or talk. The body is frequently prevented from acting out dreams upon leaving rapid eye movement (REM) sleep, a paradoxical state of sleep in which intense dreams are accompanied by total muscle paralysis. The brain awakens from REM sleep during SP before the body paralysis stops. Being awakened in complete darkness, feeling helpless and paralyzed, and unable to scream are all part of this horrific experience. The sensory flood from the dream world still obscures the person's thoughts, which could lead them to hallucinations. Sleep loss, psychological stress, or irregular sleep cycles can cause this syndrome. The primary symptom is the inability to move or talk upon awakening. Murmurs, voices, roars, and hissing, static, zapping, and buzzing noises are heard. Other signs and symptoms can include a fearful or pushed-down sensation, hypnagogic or hypnopompic experiences, breathing problems, sweating, headaches, muscle aches, or paranoia. SP involves a temporary inability to move or speak upon awakening, often with vivid hallucinations. It occurs when the muscle paralysis typical of REM sleep persists briefly into wakefulness. Factors like sleep deprivation and irregular sleep patterns influence this condition. Recent studies also highlight risk factors such as stress, psychiatric disorders, genetic predispositions, and, notably, hypertension. Despite being relatively common and linked to various medical conditions, SP remains poorly understood [1,2].

SP happens when atonia is based on REM and persists into awakening. Most SP sufferers also have vivid, multisensory, and frequently depressing dream activities during conscious paralysis. For most people, SP is a highly unpleasant experience that may not be readily understood due to the interaction of atonia and waking nightmares. Instead, patients could make sense of it using various non-medical theories. Thus, unexpectedly, SP is assumed to play a role in the development and persistence of many beliefs in the supernatural (e.g., night-time alien sightings and demon attacks) in people with otherwise routine reality tests [3]. More than 75% of SP episodes also include numerous bizarre and often terrifying hallucinations in addition to ongoing muscle atrophy. We can classify these experiences into three groups. Other elements of intruder hallucinations include a strong multisensory visual of an intruder in the bedroom and a feeling of a demonic presence in the space. A pressure-like sensation on the chest, often accompanied by suffocating or choking sensations, is a characteristic of incubus hallucinations. Both of these kinds of hallucinations typically happen simultaneously. False movement sensations, out-of-body experiences, and out-of-body autoscopy are all included in the vestibular-motor (V-M) category of hallucinations [4]. SP in isolation doesn't require regular medical treatment, whereas SP that occurs with narcolepsy does, particularly when symptoms interfere with daily job and home life. The most frequently administered drugs are stimulants and selective serotonin reuptake inhibitors (SSRIs), which help people stay awake and cure narcolepsy. Medical professionals occasionally attach electrodes to the chin, scalp, and outside edges of the eyelids to detect electrical impulses in the brain and brain waves and keep track of breathing and heart rates [1]. This methodological approach provided a structured framework for gathering relevant evidence to address the research questions regarding the relationship between SP, its risk factors, and depression in older adults.

Future research on SP should address both its neurological sequelae and potential improvements in daily routines and treatment approaches. This includes exploring cognitive and structural brain changes, optimizing sleep hygiene, developing personalized treatments, and enhancing public awareness. Understanding these aspects could lead to more effective management strategies and improved quality of life for individuals experiencing SP.

## Review

**Figure 1:**
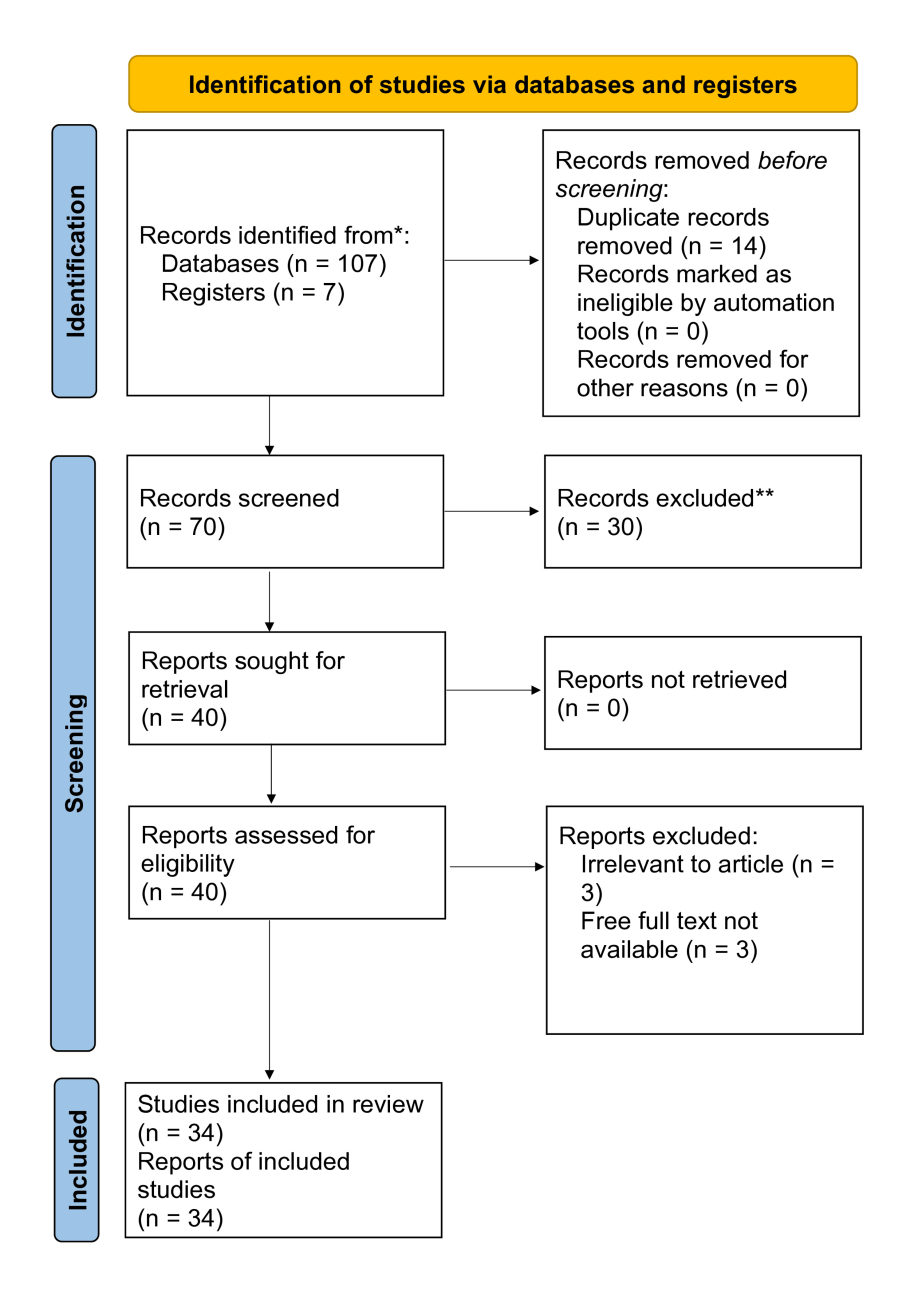
Inclusion and exclusion criteria for the article *Consider, if feasible, reporting the number of records identified from each database or register searched (rather than the total number across all databases/registers). **If automation tools were used, indicate how many records were excluded by a human and how many were excluded by automation tools.

The methodology details the systematic approach employed for a literature review on SP and its correlation with various risk factors. It involved searching three major databases, PubMed, Scopus, and Google Scholar, using Medical Subject Heading (MeSH) phrases such as "Sleep Paralysis," "Rapid Eye Movement (R.E.M.)," "Obstructive sleep apnea," and "Narcolepsy" and Boolean operators "And" to combine terms effectively. The aim was to identify published articles, studies, and research in English related to SP and its associated risk factors. Inclusion criteria were set to include studies published between 1988 and 2024 exclusively focused on depression in older adults and its risk factors. The selection process, depicted in Figure 1, likely involved screening titles, abstracts, and full texts of articles based on predefined criteria. The method used to choose the research is shown in Figure 1 below.

Discussion

SP is when voluntary muscular movements are stopped at the start of sleep or upon waking up. The local surroundings are perceived clearly, and breathing and eye movements are unaffected. These episodes are typically accompanied by a range of hallucinations, including illusory perceptions of movement (V-M hallucinations), pressure on the chest (incubus hallucinations), and a feeling of an evil entity (known as intruder hallucinations). Over 100 different cultures have terminology for SP, making it a truly global occurrence. Experiences with SP are frequently incorporated into a culture. It has been proposed that experiences of SP may be the cause of purported paranormal events like witches [2,5]. A brief partial or total paralysis at the start or end of the sleeping cycle is known as SP [6]. Aside from the limits of the techniques used to detect SP, another significant issue is a lack of consistency in terminology.

Nauseous paralysis during sleep is a typical symptom of narcolepsy. Other symptoms of narcolepsy include daytime tiredness, inconsistent nocturnal sleeping patterns, and cataplexy, which is the spontaneous, brief, bilateral loss of muscle tone in reaction to strong emotions such as laughter or anger [7,8]. In cases where episodes produce clinically substantial fear and distress, some authors sometimes refer to cases as "fearful" isolated SP (ISP). Finally, recurring (frightened) ISP may be used when episodes reoccur. However, no agreement exists about how frequently episodes must occur to be termed recurrent [9].

A recent systematic review of 35 research studies, encompassing a total of 36,533 individuals, found that 7.6% of the population had experienced at least one episode of SP in their lifetime. Students (28.3%) and psychological patients (31.9%) reported higher lifetime incidence percentages of SP, with slightly more women than men reporting it [3,10]. In addition to being associated with conditions such as narcolepsy, hypertension, and seizure disorders, SP episodes have also been linked to shift work, generalized sleep deprivation, airline delays, student status, and African ancestry. When SP occurs in otherwise healthy individuals, it is called ISP. It is no longer recognized that SP and ISP episodes are diagnosable illnesses [2,10]. Numerous studies have demonstrated a connection between poor sleep quality and an increased likelihood of experiencing SP. SP has been associated with symptoms of insomnia, though not with true insomnia. Research also indicates a connection between SP and various strange and frightening sleep experiences, including nightmares, "expanding syndrome," and "lucid dreaming." According to the objective tests, SP is a "mixed" state of consciousness that blends aspects of REM sleep and wakefulness [11].

Risk factors for SP

Several risk factors have been discovered, in addition to the demographic indicators linked to SP that were previously listed. Table 1 lists some medical disorders connected to SP.

**Table 1:** Several medical disorders associated with sleep paralysis REM: rapid eye movement

S. no.	Factors	Description
01	Hypertension	An individual has symptoms brought on by adrenergic stimulation following an isolated episode of sleep paralysis (e.g., tachycardia, sweating, tremulousness, panic). Solitary sleep paralysis seems to be brought on by sleep-wake cycle dysrhythmia, which is partly regulated by adrenergic processes. This data implies that isolated sleep paralysis involves adrenergic dysfunction. Delirium tremens, a withdrawal syndrome caused by overactive locus coeruleus and marked by hypertension and symptoms of anxiety, provides additional proof in favor of the association between stress, adrenergic overactivity, and high blood pressure [12].
02	Obstructive sleep apnea	The most common respiratory issue linked to sleep is obstructive sleep apnea. People who have obstructive sleep apnea continually stop and start breathing. Obstructive sleep apnea, a type of sleep disorder, involves recurrent airway blockages caused by relaxed throat muscles, often occurring intermittently during sleep. Snoring commonly accompanies obstructive sleep apnea as a symptom. Another alternative is using a mouthpiece to advance the lower jaw during sleep (e.g., loud snoring, morning headache, high blood pressure, waking during the night, and gasping or choking) [13].
03	Idiopathic hypertension	Idiopathic hypertension is responsible for the majority of cases of high blood pressure. There's a longstanding theory suggesting that higher salt consumption increases the likelihood of hypertension. Essential hypertension in people has been linked to a genetic susceptibility to salt sensitivity. Approximately 50-60% of individuals exhibit this sensitivity to salt, which heightens their risk of developing hypertension. Coronary heart disease, stroke (both ischemic and intracranial), myocardial infarction, and hypertensive encephalopathy are all conditions that can be caused by or associated with sleep paralysis.
04	Narcolepsy	Hypnagogic experiences are not uncommon in narcolepsy, a neurological disorder affecting approximately one in 1,000 individuals. This condition disrupts standard sleep patterns, leading to irregular sleep cycles. Symptoms associated with narcolepsy include abnormal manifestations of REM sleep, such as sleep paralysis, cataplexy, hypnagogic hallucinations, anomalous sleep-onset REM periods, and interrupted nocturnal sleep. Many social issues, including marital conflict and fatal car accidents, have been linked to excessive daytime sleepiness [15].
05	Insufficient sleep syndrome	Insufficient sleep syndrome, known as sleep deprivation, arises primarily from individuals' sleep habits rather than underlying medical, emotional, or environmental factors. This means inadequate rest occurs due to lifestyle choices rather than external influences. Sleep deprivation results when people prioritize other activities over sufficient rest. Insufficient sleep syndrome causes emotional disturbances and affects the brain's functions, the brain's structure, and the cardiovascular system [16,17].
06	Alcohol use	Alcohol consumption can lead to excessive daytime sleepiness and unexpected sleep attacks, which are common symptoms of alcohol-induced narcolepsy. Consuming alcohol can cause sleep paralysis and disturbance. Alcohol consumption and withdrawal can affect REM sleep, potentially leading to sleep disorder disorders like sleep paralysis. Similarly, certain medications and substances, such as nicotine, can occasionally trigger episodes of sleep disturbance [9,18].
07	Wilson's disease	Wilson's disease is a sporadic genetic illness that leads to the accumulation of copper in various organs, such as the liver, brain, and eyes. While it can affect individuals of any age, it is typically diagnosed in those aged between five and 35. Copper plays a vital role in forming strong bones, collagen fibers, nerves, and the skin pigment melanin. Copper is obtained from the food we consume, and any excess copper is eliminated from the body through the liver's bile production, which is directly linked to sleep paralysis. Symptoms of Wilson's disease include difficulties in swallowing, speaking, or coordinating physical movements, depression, mood swings, insomnia, and involuntary muscle movements or stiffness [19].

SP occurs frequently in the general population and has a unique and dramatic clinical appearance. Many writers, such as Fyodor Dostoevsky, say that SP is characterized by a unique combination of subjective wakefulness and total weakness, distinguishing it from situations where individuals feel sluggish or heavy. During an episode of SP, individuals typically experience a sense of being fully awake but unable to move, which can be extremely frightening, particularly during the first occurrence. This feeling is often accompanied by sensations of pressure on the chest or a heavy weight on the rib cage. These sensations arise because not only are the auxiliary respiratory muscles (intercostal muscles) affected but also the voluntary limb muscles. However, it's important to note that the diaphragm responsible for breathing remains unaffected during SP episodes. This combination of subjective wakefulness, paralysis, and sensations of pressure or weight can contribute to the distressing nature of SP experiences [20,21]. If a person with high blood pressure chose not to go through ISP, they were very likely to have panic episodes often, leading to a diagnosis of panic disorder. This situation was similar to finding out that none of the family members with panic disorder admitted to experiencing ISP [12]. It briefly mentions that people experiencing SP might hear or see voices, leading them to believe they are being attacked. It was reported that the depressing emotions experienced by these patients frequently lingered throughout the next day as acute depression [22]. After reviewing the body of research on lifetime episodes of SP, it was found to be a relatively common occurrence. It is notably more prevalent among students and individuals with mental health conditions, with a minimal discrepancy between these two groups despite occurring in less than 8.0% of the general population. While the reasons behind this higher prevalence remain uncertain, regular sleep issues experienced by both groups likely contribute to the likelihood of experiencing SP episodes [10].

Patients who report experiencing atonic muscles during sleep often overlook this common symptom despite experiencing significant daytime sleepiness that interferes with their daily lives. Polysomnography typically yields unremarkable results, except for scattered nocturnal sleep with decreased REM sleep. Consequently, to confirm a diagnosis of narcolepsy, multiple sleep latency tests (MSLT) are conducted. Subsequently, patients may commence treatment with prescription drugs such as stimulants and antidepressants. For instance, venlafaxine, administered once daily, has been found effective in alleviating cataplexy and SP. However, research investigating the efficacy of specific medications in treating SP is limited [23,24]. Hence, one possible explanation for this correlation could be that individuals experiencing sleep-related auditory or visual hallucinations may interpret them as evidence of the existence of aliens or other supernatural entities. Nevertheless, additional research is necessary to validate this hypothesis and eliminate alternative explanations [25]. The individuals experienced multimodal hallucinations, with many of their encounters aligning with Cheyne-Stokes respirations [26,27].

Alprazolam patients experience improved SP due to its anxiolytic properties, which promote a sense of ease and facilitate a regular, healthy sleep cycle. Similarly, chlordiazepoxide, an anticonvulsant and short-term anxiety medication used to treat alcohol withdrawal symptoms, enhances postural atonia and encourages a regular sleep cycle. The recommendations are based on narcolepsy research, limited case studies, clinical expertise, and logical deductions derived from the key findings on SP and recurrent isolated sleep paralysis (RISP). Fortunately, various practical treatment approaches exist in psychopharmacology and psychotherapy [3]. To prevent and manage SP, individuals should maintain a consistent sleep schedule, aim for 7-9 hours of quality sleep, and manage stress with relaxation techniques. Optimizing the sleep environment by keeping it dark, cool, and quiet, avoiding stimulants like caffeine and alcohol before bed, and sleeping on your side can also help. For ongoing issues, consulting a healthcare professional is recommended. It notes the inclusion of an adherence measure to aid research, although the manual lacks empirical validation. Cognitive-behavioral therapy for ISP involves tailoring sleep hygiene advice to the individual and teaching relaxation methods specifically designed to manage recurring episodes of RISP, in vivo interventions to disrupt episodes, strategies for coping with alarming hallucinations, challenging catastrophic thoughts, and simulating successful resolutions of RISP episodes [28].

Multiple hallucinations are often associated with SP. One component, known as "intruder hallucination," involves feelings of panic, a sense of presence, and both auditory and visual hallucinations. Another component is the incubus hallucination, characterized by chest pressure. Lastly, out-of-body experiences are characterized by V-M hallucinations. ISP is the temporary inability to move from sleep to wakefulness without other clinical signs of narcolepsy. ISP episodes are more commonly linked to hypnagogic events than narcolepsy-associated paralysis, which typically occurs upon awakening from hypnopompic paralysis [29]. During SP, hallucinations occurring during the onset of sleep (hypnagogic) or upon waking (hypnopompic) can manifest in various forms. These include seeing human-like shapes approaching, hearing footsteps, feeling levitation, experiencing autoscopy (out-of-body sensations), or perceiving the presence of a frightening intruder through auditory, tactile, or visual sensations. Specifically, individuals experiencing SP often report hallucinations of a shadowy, human-like figure exerting pressure on their chests as if suffocating them. These universal aspects of the SP experience reported globally across different cultures may stem from underlying neurobiological factors (Hufford, 1982, 1995, 2005). Not surprisingly, SP frequently triggers intense panic and fear in those who experience it [30-32].

Table 2 shows a summary of the study findings included in this review.

**Table 2:** Summary of study findings PTSD: post-traumatic stress disorder; RBF: random bits forest; SVM: support vector machine; REM: rapid eye movement; CNS: central nervous system; RISP: recurrent isolated sleep paralysis; ISP: isolated sleep paralysis

Author	Years	Findings
Goel [1]	2022	Sexually abused individuals often experience sleep paralysis, closely tied to their trauma. This phenomenon involves a sense of wakefulness alongside physical immobility, occurring during the transition between wakefulness and sleep.
Sateia [2]	2014	There were seven significant categories of sleep disorders: insomnia disorders, disorders related to breathing during sleep, central disorders causing excessive daytime sleepiness, circadian rhythm sleep-wake disorders, disorders involving abnormal movements during sleep, parasomnias, and miscellaneous sleep disorders.
Sharpless [3]	2016	The available treatment options were reviewed after discussing these topics and offering suggestions for accurate diagnosis, differential diagnosis, and patient selection. These options encompass pharmacological and psychotherapeutic interventions, which, though promising, necessitate further empirical support and more extensive, well-controlled trials.
Denis [4]	2018	Research shows a correlation between poorer sleep quality and an increased likelihood of experiencing sleep paralysis. Symptoms of insomnia, rather than a diagnosed insomnia disorder, can also predict episodes of sleep paralysis.
Denis et al. [5]	2017	Studies extracted data on sleep paralysis measures and analyzed relationships with various linked variables. Factors such as drug abuse, stress, trauma, genetics, physical health issues, personality traits, IQ, unusual beliefs, subjective and objective sleep disturbances, anxiety symptoms in non-clinical samples, and mental disorders were identified. Sleep paralysis was notably prevalent in individuals with PTSD and, to a lesser extent, panic disorder.
Mahlios et al. [8]	2013	Narcolepsy results from the loss of orexin (also known as hypocretin) neurons in the lateral hypothalamus. Evidence strongly supports an autoimmune mechanism that targets and destroys these neurons.
Sharpless and Barber [10]	2011	The study identified a significant relationship between age and sleep paralysis status. Future research should consistently and comprehensively disclose essential demographic data, emphasizing understanding variations in prevalence rates across different ethnicities.
Akhtar and Feng [11]	2022	Poor sleep quality correlates with sleep paralysis. Among the models tested on the dataset, the random forest achieved the highest prediction accuracy for sleep paralysis at 91.9%. Polynomial achieved an accuracy of 47.56%, RBF achieved 42.68%, and SVM with linear kernel achieved 80.49%.
Bell et al. [12]	1988	In a preliminary study of 31 individuals diagnosed with hypertension, 41.9% reported experiencing isolated sleep paralysis, 35.5% reported having panic attacks, and 9.7% were diagnosed with panic disorder.
Mitler et al. [15]	1990	Narcolepsy, a neurological disorder affecting up to one in 1,000 people, involves irresistible sleep episodes and features like REM sleep manifestations (cataplexy, sleep paralysis, hallucinations). It's linked to HLA-DR2 and DQw1. Treatment includes CNS stimulants for sleepiness and tricyclics for REM abnormalities. Studies show methylphenidate and dextroamphetamine effectively reduce sleepiness, pemoline enhances performance, and protriptyline and viloxazine help with cataplexy more than sleepiness.
Chattu et al. [17]	2018	Recent decades saw decreased sleep duration, linked globally to health risks, higher mortality rates, impaired cognition, and productivity. There is an urgent need for ISS awareness, economic analysis, and policy as a noncommunicable disease.
Wróbel-Knybel et al. [18]	2022	Our study reveals that a significant number of students experienced isolated sleep paralysis, often influenced by lifestyle choices, mental health conditions, and physical health factors. Sleep paralysis is influenced by risk factors such as sleep deprivation, irregular sleep patterns, and high stress or anxiety. It can also be affected by conditions like narcolepsy and insomnia, as well as sleeping on your back. Excessive alcohol use, certain medications, and mental health issues like depression further increase the risk of sleep paralysis.
Ali et al. [20]	2009	Most patients with idiopathic hypersomnia show positive responses to treatment. Despite being less commonly used initially, methylphenidate is favored over modafinil as the final monotherapy for managing idiopathic hypersomnia.
Anderson et al. [21]	2007	It was not feasible to predict the severity of the condition or the response to treatment based on the result of the sleep latency test. The results showed that clinical characteristics varied widely in intensity and were heterogeneous.
Stores [22]	1998	The findings stress the importance of distinguishing sleep paralysis from mental illnesses and other sleep issues, as it can lead to severe and unpleasant hallucinations.
Choi et al. [23]	2018	Sleep paralysis, characterized by REM sleep-related muscle atonia during wakefulness, was observed in two narcolepsy patients aged 22 and 19. Clinicians assess factors like stress, trauma, genetics, physical health, sleep disorders, and psychiatric conditions in evaluating sleep paralysis cases. Its prevalence among narcolepsy patients is 20-50%, underscoring the importance of assessing narcoleptic symptoms and performing multiple sleep latency tests for accurate diagnosis in such cases.
Rauf et al. [25]	2023	The findings suggest connections between beliefs in the paranormal and different sleep factors. This knowledge could enhance our ability to improve sleep through psychoeducational approaches.
Lišková et al. [27]	2016	The findings showed that the average age of first sleep paralysis occurrence was 17 years, with 24% of respondents reporting sleep paralysis starting before age 15. It was proposed that sleep paralysis among university students is not typically caused by irregular sleep patterns or sleep deprivation, which are common factors in this demographic.
Terrillon and Marques-Bonham [28]	2001	The findings indicate that more than 90% of respondents feel intense fear during their episodes of RISP. Approximately 50% attribute these episodes to paranormal or supernatural causes. A typical RISP episode is characterized by three main components. Finally, we provide a concise overview of potential coping strategies for RISP.
Mainieri et al. [29]	2021	During sleep paralysis, the electroencephalography spectrum displayed intermediate characteristics between wakefulness and REM sleep in the alpha, theta, and delta frequencies. However, there was no significant difference in beta frequencies between sleep paralysis and normal REM sleep. The power spectrum during false awakening exhibited a similar pattern to that observed during sleep paralysis.
Jalal et al. [30]	2015	Case studies are provided to exemplify the study's findings. The Pandafeche attack represents a culturally specific, supernatural interpretation of the phenomenon of sleep paralysis in the Abruzzo region of Italy.
Jalal et al. [31]	2021	Thirty-seven percent of participants used supernatural and religious methods like dua (prayer), reciting the Quran, and wearing a musqa (talisman inscribed with Quranic verses) to prevent future sleep paralysis attacks. Case studies are presented to demonstrate these findings. The Karabasan represents a culturally specific, supernatural interpretation of sleep paralysis in Turkey.
Takeuchi et al. [32]	2002	Hit rates in the vigilance task remained steady in non-ISP episodes but declined in later stages for ISP episodes. Subjective sleepiness decreased over time for non-ISP episodes but slightly increased for ISP episodes.

During sleep, the body cycles through REM and non-rapid eye movement (NREM) stages. REM sleep involves heightened brain activity, increased heart rate and blood pressure, and faster breathing. Vivid, detailed dreams and occasional nightmares characterize it. Interestingly, REM sleep induces muscular atonia, a temporary paralysis likely to prevent injury from dream-related movements. SP blurs the line between sleeping and waking states. It involves temporary muscle paralysis lasting seconds to minutes, during which individuals are conscious but unable to move. They can breathe, think, and see, yet remain physically immobile. SP may include hallucinations, where individuals perceive unreal alterations in their environment. These can range from simple images to complex sensory experiences like intruder, incubus, or V-M hallucinations [33].

## Conclusions

The study's findings indicate that SP occurs when an individual wakes from deep sleep but is temporarily unable to move or speak. This phenomenon is largely attributed to continuous muscle paralysis associated with REM sleep, a phase characterized by vivid dreaming and complete muscle immobilization. Contributing factors include sedentary lifestyles, which are known to exacerbate this condition, and the specific characteristics of an individual's working environment, which can influence both the frequency and severity of SP episodes.
